# Stimulation of the Vascular Endothelium and Angiogenesis by Blood-Flow-Restricted Exercise

**DOI:** 10.3390/ijerph192315859

**Published:** 2022-11-29

**Authors:** Mikołaj Maga, Martyna Schönborn, Agnieszka Wachsmann-Maga, Agnieszka Śliwka, Jakub Krężel, Aleksandra Włodarczyk, Marta Olszewska, Roman Nowobilski

**Affiliations:** 1 Department of Rehabilitation in Internal Diseases, Faculty of Health Sciences, Jagiellonian University Medical College, 31-066 Krakow, Poland; 2 Clinical Department of Angiology, University Hospital in Krakow, 30-688 Krakow, Poland; 3 Department of Angiology, Faculty of Medicine, Jagiellonian University Medical College, 30-688 Krakow, Poland; 4 Department of Pediatrics, Institute of Pediatrics, Faculty of Medicine, Jagiellonian University Medical College, 30-663 Krakow, Poland

**Keywords:** blood flow-restriction, vascular functions, angiogenesis factor, physiotherapy techniques

## Abstract

Blood-flow-restricted exercise (BFRE) has been gaining constantly increasing interest in rehabilitation, but its influence on endothelial functions has not been well studied yet. Our aim is to examine the influence of low-resistance BFRE on endothelial functions and angiogenesis. This prospective cross-over study involved 35 young healthy adults. They conducted a 21-min low-resistant exercise with blood flow restricted by pressure cuffs placed on arms and tights. They also did the same training but without blood flow restriction. Endothelial parameters and angiogenesis biomarkers were evaluated before and up to 20 min after exercise. Both types of exercise increased Flow-Mediated Dilatation (FMD) but elevation after BFRE was more significant compared to the controls. The stiffness index decreased only after BFRE, while the reflection index decreased significantly after both types of exercise but was higher after BFRE. Platelet endothelial cell adhesion molecule (PECAM-1) and vascular endothelial growth factor receptor 2 (VEGFR-2) concentrations were increased by both exercise types but elevations were higher after BFRE compared to the controls. Only BFRE elevated the mean serum CD34 protein concentration. Based on these results, we can assume that low-resistance BFR exercise stimulates angiogenesis and improves endothelial functions more significantly compared to the same training performed without blood flow restriction.

## 1. Introduction

Over the past decade, blood-flow-restricted exercise (BFRE) has been gaining constantly increasing research interest [1]. It originates from KAATSU, described as an exercise practice with mechanically restricted blood flow performed by placing flexible pressure cuffs around the upper or lower extremities proximal to the working muscle [2,3]. Modern BFRE is based on venous blood flow obstruction, leading to passive congestion without causing ischemia [4].

The range of BFRE exercises includes low-intensity as well as high-intensity workloads and varies in multiple different types of exercise, such as walking, running, cycling, weight lifting, hand grip, yoga, or cross-training [5,6,7]. Multiple studies have reported that BFRE induces hypoxic stress in skeletal muscles which causes muscle hypertrophy and increases not only muscle strength but also endurance [8,9]. Some studies have raised an important question on how BFRE influences vascular functions but did not produce conclusive results [10,11,12]. The main hypothesis assumes that the effect of reactive hyperemic blood flow caused by BFRE-induced increased shear stress led to vasodilatation and stimulation of endothelial-factor production. This could result in an improvement of endothelial functions and a significant angiogenesis enhancement [11]. Although BFRE is still seen more as a novelty than a commonly used exercise method, its role in vasodilatory regulation and influence on endothelial functions needs to be examined and clearly explained.

Endothelial dysfunctions play a crucial role in the atherosclerosis processes since the endothelium is the main regulator of vascular-wall homeostasis. Due to multiple factors, it is constantly modulated into a nonadaptive state, including dysregulation or even loss of the homeostatic mechanisms. This results in increased expression of adhesion molecules stimulating the synthesis of pro-inflammatory and prothrombotic factors, oxidative stress leading to impaired endothelial vasodilation, and the development of atherosclerotic plaque development [13,14]. For patients in an early phase of peripheral arterial disease, the angiogenic process can lower the level of ischemia by providing new blood flow pathways to tissues. This phenomenon leads to a reduction in ischemic symptoms: pain reduction, an extension of claudication distance, or even ulceration healing [15]. While the impact of percutaneous transluminal angioplasty (PTA) with or without stenting remains unclear [16,17], lifestyle modifications, including smoking cessation, proper diet, and regular physical activity, are some of the most efficient non-invasive methods of endothelial stimuli [18]. Non-invasive methods of treating patients with PAD, which can improve their quality of life, are constantly sought. However, although physical training with restricted blood flow is relatively well known, its use in medicine has not been well studied, especially in the aspect of improving endothelial functions, which could bring many benefits for PAD patients.

If proven to stimulate angiogenesis and improve endothelial functions, it may be expected for BFRE to serve as a useful means in rehabilitation, by physical exercise, of noncritical PAD patients.

Therefore, the objective of this study was to compare the short-term endothelial and angiogenic response between blood-flow-restricted exercise and the same exercise without blood-flow restriction in young, physically active, healthy adults.

## 2. Materials and Methods

### 2.1. Study Design

**Figure 1:**
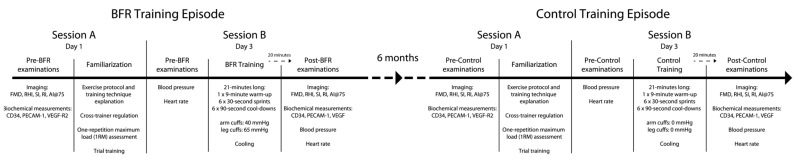
Study timeline.

This was a prospective cross-over randomized study (Figure 1). It consisted of 2 separate training episodes: one that included the blood-flow-restricted exercise (BFRE episode); the second with the same exercise but without restriction of blood flow (control training, CT episode).

The study involved healthy adult volunteers with normal BMI of 18–30 years old. The exclusion criteria were: a physical disability that prevents exercise in accordance with protocol, professional/competitive sports activities, or any comorbidities.

### 2.2. BFR Exercise and Control Training Protocols

Each participant exercised in both the BFRE and CT episodes carried out 6 months later in random sequence. During the first training day (Session A) of each episode, physical and imaging examinations were performed and blood samples were taken. Then, all participants took part in familiarization with the exercise protocol and proper training technique, including regulations of handgrip length and seat position of a cross-trainer. The cross-trainer was equipped with professional software that, combined with the pulse detector, not only assessed the proper one-repetition maximum load (1RM) but also allowed to choose any desired 1RM percentage to warm-up, sprint, as well as for cool-down periods of training and to keep it during the whole session. It was used during the familiarization session to assess the 20% 1RM for every participant, and as a sprint resistance for Session B. Two days later (Session B), each participant completed a full 21-min exercise. Up to 20 min after physical effort, laboratory and physical measurements were performed once again. Endothelium imaging was performed 60 min after exercise. Before and after exercise, blood pressure, heart rate, and oxygen saturation were measured, accompanied by assessment of dyspnea and fatigue intensity using a modified 0–10 Borg Scale.

All participants were instructed not to perform any physical activity other than regular daily activity for 72 h prior to study exercises and measurements as well as between Sessions A and B. They were also required not to use bicycles as a means of transport and recommended private or public transport instead. Measurements, as well as examinations and blood sampling, were conducted at the same part of the day in every case. An interval 21-min-long exercise was performed on NuStep T5XR cross-trainer consisting of 9-min warm-up (10% 1RM), 6 × 30-s-long sprints with increased resistance (20% 1RM), separated by 90-s cool-downs with lower resistance (10% 1RM). The resistance load was set individually and presented on the display as a graph during training to help the participants maintain the steady pace of exercise. In BFRE, vein occlusion was achieved by the use of cooling liquid pressure cuffs (arm cuffs with 40 mmHg and leg cuffs with 65 mmHg) provided by the VasperTM system and accompanied by a cooling seat and kept for the whole duration of the session [19]. In the CT, the same exercise protocol was carried out with the same exercise tools as for BFRE, however, the pressure cuffs were not inflated and therefore vein occlusion did not occur.

### 2.3. Endothelial Parameters Measurements

Endothelial functions were assessed by non-invasive imaging examinations including flow-mediated dilatation (FMD), reactive-hyperemia index (RHI), and arterial pulse-waveform analysis (aPWA), which were performed in Session A as well as Session B for both BFRE and CT episodes:

- FMD: Current guidelines for the ultrasound assessment of FMD were used in the study [20]. Before the test, the participants were resting in a supine position. FMD was measured with the Siemens Acuson S2000;
- RHI: Reactive hyperemia index (RHI) was measured using Peripheral Arterial Tone (PAT) analysis on Itamar Medical Endo-PAT 2000 device to assess digital pulse volume changes. The digital pulse amplitude was recorded continuously during the test and recorded on a laptop. A computerized algorithm provided by Itamar Medical was used to analyze and automatically calculate the RHI;
- aPWA: Arterial pulse waveform analysis (aPWA) was performed by recording 20 peripheral pressure waveforms from the radial artery at the wrist using applanation tonometry with a high-fidelity micro manometer, followed by generating the corresponding central aortic pressure waveform. The arterial stiffness evaluation enabled a commercially available SphygmoCor system (AtCor Medical). Stiffness index (SI), reflection index (RI), and corrected augmentation index (AI75) were measured to assess large, medium-sized, and small arterial stiffness variables.

### 2.4. Angiogenesis Assessment

To monitor angiogenic processes, blood samples were collected before and up to 20–30 min after exercise. They were centrifuged for 15 min with a rotation speed of 2000 rotations per min and kept frozen at −80 °C. Measurements of vascular endothelial growth factor receptor 2 (VEGF-R2) and stem cells: serum levels (platelet endothelial cell adhesion molecule PECAM-1 and CD34) were made. The enzyme-linked immunosorbent assay (ELISA) method was used as a determining method.

### 2.5. Statistical Analysis

Statistical analysis was performed using Statistica software, version 13.1 (TIBCO Software Inc., Palo Alto, CA, USA). The sample size of 35 participants was calculated with a power of 0.9 and a significance level of 0.10. We did not assume any lost-to-follow-up participants. The Shapiro-Wilk test was applied to confirm the normal distribution of the data. The results were presented based on the parameters of descriptive statistics, including numbers with percentages for categorical variables and either mean values and standard deviations, or median values with quartiles for continuous variables. To compare the response of BFRE and the CT, the Friedman rank test and repeated measures ANOVA with Greenhouse–Geisser correction were performed. For post hoc multiple comparisons, Tukey’s and Dunn’s tests were used. *p*-values < 0.05 were considered statistically significant.

### 2.6. Ethical Aspects

The work described in this article was carried out in accordance with the Code of Ethics of the World Medical Association (Declaration of Helsinki) for experiments involving humans and the proper consent for this study was obtained from the constituted committee for human subjects or animal research at Jagiellonian University Medical College. Participants were informed about the purposes, risks, and discomforts associated with the study. All participants gave their voluntary written consent to participate in the study.

## 3. Results

### 3.1. Participant Population

Thirty-five healthy participants were enrolled in the study, 45.71% of them were men, aged 24.29 years (±2.44), with a median BMI of 21.26 (19.95–23.89). None of them were a current smoker, one person had a history of smoking but ceased smoking over 12 months prior to the study and had a record of only 0.5 smoking pack-years. They did not have any comorbidities. Although none of the participants were a professional or competitive sportsman/sportswoman, two participants reported playing squash frequently once a week and three were jogging up to four times per week.

### 3.2. Endothelial Response to BFR Exercise

**Figure 2:**
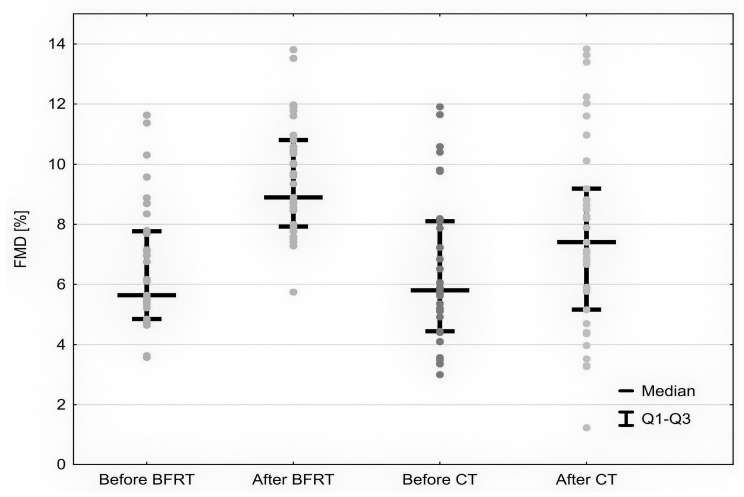
Comparison of FMD changes between participants after BFRE and control training.

The median FMD, compared to baseline values, increased significantly after BFRE (to 8.9% (7.93–10.8) from 5.64% (4.85–7.77), *p* < 0.05) as well as after control training (to 7.41% (5.16–9.18) from 6.38% (4.44–8.1), *p* < 0.05) but the mean ΔFMD was greater after BFRE compared to control training (+2.89% vs. +1.33%, *p* < 0.001) (Figure 2). The reactive hyperemia index (RHI) remained unchanged after BFRE (1.85 vs. 1.9, *p* = 0.92) and control training (1.82 vs. 1.92, *p* = 0.93).

Although SI after the BFRE was insignificantly lower compared to after CT (7.01 vs. 7.4) and the ΔSI_BFR_ was insignificantly higher than ΔSI_control_ (−0.19 vs. +0.04, *p* = 0.07), the trend was the opposite: after the BFRE, SI decreased, while after control training it increased (from 7.21 to 7.01 vs. from 7.34 to 7.4). In addition, although the average RI decreased after both types of exercise—BFRE (from 70.86 ± 9.69 to 63.43 ± 9.46, *p* < 0.001) and control training (from 71.89 ± 8.99 to 69.49 ± 7.92, *p* = 0.08)—the decrease was greater after BFRE compared to control training: ΔRI_BFR_ 4.0 (1.0–5.0) vs. ΔRI_control_ 2.0 (−1.0–6.0), *p* < 0.001. AI75 was not significantly influenced by BFRE (from −18.0 to −16.0, *p* > 0.05) nor by the control training (from −18.0 to −15.0, *p* > 0.05). 

### 3.3. Angiogenesis

**Figure 3:**
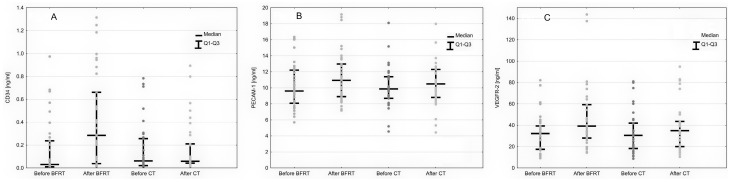
Changes in CD34 (**A**), PECAM1 (**B**) and VEGFR-2 (**C**) compared between participants after BFR exercise and control training.

The serum PECAM-1 concentration was increased by both types of training (BFRE: from 9.61 ng/mL to 10.94 ng/mL; CT: from 9.87 ng/mL to 10.49 ng/mL), but the change was significantly higher in the BFRE than in control training (ΔPECAM-1_BFR_ vs. ΔPECAM-1_control_: 1.11 ± 0.83 vs. 0.37 ± 0.55, *p* < 0.001) (Figure 3B). 

Only BFRE influenced the serum CD34 concentration by increasing it (from 0.03 ng/mL to 0.28 ng/mL, *p* < 0.05) when it remained unchanged by control training (0.062 ng/mL to 0.059 ng/mL, *p* > 0.05) (Figure 3A). 

Serum VEGF-R2 concentrations were increased by both types of training [BFRE: from 32.2 ng/mL to 39.19 ng/mL, *p* < 0.005; CT: from 30.46 ng/mL to 35.01 ng/mL, *p* < 0.005) but the elevation after BFRE was significantly higher compared to control training [ΔVEGF-R2BFR: 6.35 (1.83–21.72) vs. ΔVEGF-R2control: 2.86 (1.21–7.06), *p* < 0.001) (Figure 3C).

## 4. Discussion

Physical training is well recognized as a factor influencing endothelial functions [21]. However, although most physical activities benefit in improving endothelial vasodilatation abilities by increasing FMD, the matter of vascular stiffness and the perfect balance between intensity, type of training, and endothelial responses remains unsettled [22]. It has been proved that regular aerobic exercises decrease the thickness of the intima-media complex and reduce stiffness indexes in both healthy as well as cardiovascular patients [23,24]. 

However, blood flow restriction training is a well-known factor influencing muscle strength, and the mechanisms that lead to a hypertrophic response remain unclear [25]. There is also some serious concern about BFRE, especially regarding the original KAATSU exercise methods. There are multiple definitions of BFR, which include different types of occlusive tools that result in different influences on the vessels and muscles [26]. The degree of compression spread from 50 mmHg [27] up to even 300 mmHg [6]. In most of the studies, the compression was set fixed, and not related to the systolic and diastolic blood pressure of the participants. Therefore, Spranger M. et al. raise an important question of whether the current state of knowledge is solid enough to introduce BFRE into the rehabilitation of patients, including those suffering from PAD [28]. 

Our results show that BFR exercise, as well as regular cross-trainer-based training, influence the endothelium by improving its vasodilatory functions, but BFRE has a greater impact on its functions. The improvement in FMD was observed after both forms of training but its scale differed significantly. In most of the previously published studies, this parameter was not affected by BFR differently compared to regular activity [6,10,29]. Only two studies demonstrated the changes in FMD after blood flow restriction during exercises, but it was its reduction, which is opposite to our results [30,31]. As it was observed only just after the activity and it was not present 1 h later and in both cases involved the handgrip exercise, we assume that vasodilatation could be impaired temporarily and the effect was not permanent. To avoid such bias, in our study the FMD measurement was carried out 60 min after the acute effects were no longer significant. 

Although arterial stiffness is one of the most crucial parameters of the vascular wall [32], it was not directly examined after performing BFRE. Only a few published results are based on compliance of the large (LAEI) and small (SAEI) arteries, which are the surrogates of arterial stiffness based on blood pressure measurements, and did not show any significant changes [33,34]. The same studies involved analysis of systemic vascular resistance (SVR) but the results were not conclusive. The reason could be the small study population of 18 patients combined, but also the fact that BFR was combined with a vibration platform. As the examination was performed just after training, the pulse wave analysis could be influenced by this type of factor. Our study involved a larger population, included a two-parameter evaluation of the reflexion and stiffness indexes, and was conducted 60 min after the end of the training, giving sufficient time for the vessel wall to avoid examining the temporary acute effect. Although the differences in RI and SI were not statistically significant, the trend of their reduction was observed and the change was greater after BFRE compared to CT. 

The reactive hyperemia index is related to the microvascular system and its endothelial vasodilation abilities [35]. To date, the changes in RHI after BFR training were only studied once, in the elderly population and they increased after this type of activity more significantly compared to regular activity [36]. The lack of change in RHI in our study could be caused by the study design (only a single session) and the fact that participants were healthy young people; their baseline result was already correct, making it difficult to improve. 

There are several studies that address the impact of BFR on angiogenesis but in all cases, only the concentration of vascular endothelial growth factor (VEGF) was measured [36,37,38]. An elevated nitric monoxide was also observed after exercises with blood flow restriction [39]. In our study, we focused on the VEGF receptor 2 (VEGF-R2) rather than VEGF itself, as this is crucial in angiogenic mediation as signaling and represents a more durable stimulation of angiogenesis [40]. Its combination with PECAM-1 and CD-34 gives a wider perspective on the influence of BFR exercising on the process of formation of new blood vessels. While the VEGF-R2 and PECAM-1 concentrations were influenced by both forms of training (the BFR had a more significant impact), the level of CD34 increased only in the study group. As the CD34 molecule is the key factor in the regeneration of the vascular wall process [41], obtained results show the benefit of BFR over common training in patients with injured endothelium. This phenomenon was studied for the first time and opens a new pathway for the implementation of BFR exercising in patients with an impaired endothelium such as peripheral arterial disease. 

In previous studies, the reported results of the effect of BFRE on endothelium were inconclusive, mainly due to the questionable methodology: small groups, heterogeneous populations, differences in training intensity, cross-over designs, or even a lack of control groups [4,10,42]. Additionally, the most common type of training was high-load resistance, which, when combined with BFRE, often led to increased arterial stiffness [43]. Another issue is the inconsistency in the definitions of BFR, as there are multiple studies involving the primary KAATSU ischemic pressure values, which are incomparable with the blood flow restrictions based on impaired vein outflow and cannot be used in patients with impaired blood flow in their extremities [10,34,44]. 

In our study, participants performed exactly the same exercise type using the same training equipment and the same exercise protocol, which differs only by the presence or lack of blood flow restriction, to be sure that the results were not influenced in any way by changing the training protocols. The level of blood obstruction was set low enough not to limit arterial blood flow, only to restrict outflow and induce passive hyperemia. 

We decided to examine endothelial parameters by imaging methods. Measurement of the flow-mediated dilatation in the brachial artery is one of the most studied methods of vasodilatory function assessments and a recommended one [20]. Multiple studies have shown that the reactive hyperemia assessment may also be a solid and reliable endothelial parameter in an examination of vasodilatation, while at the same time is less exposed to the human-bias risk compared to FMD [45]. The stiffness parameters such as SI, RI, and AI were based on computerized averaging and the multiple pulse waveforms analysis at the baseline examination in the occluded arm. AI@75 is the corrected AI, standardized to a pulse of 75 bits per minute, and has been shown to be more reliable compared to standard AI [46]. Such selection of different imaging methods provides information not only on vasodilation values but also on the factors that cause them. 

This study has some limitations, which might have slightly influenced the results. Although the sample size was relatively small in our study, the number of participants was large enough to observe statistically significant dependencies and correlations. Nevertheless, due to the small study group planned, we decided on a cross-over study design rather than RCT, which reduces the risk of bias caused by differences between the groups of participants. Furthermore, only nonsmoking patients were enrolled, to reduce the level of carboxyhemoglobin as a confounding factor. The study was randomized on the matter of exercise-type sequences. In this study, we measured only the response to the single session BFRE and regular low-intensity training, therefore the conclusions are limited to this type of stimulation only. There is a clear need for further studies, including a longer exercise period and observation, to draw conclusions on the effects of BFRE on vascular functions in long-term exposure. 

To our knowledge, this is the first study that refers to the influence of BFRE on both endothelial functions and angiogenesis stimulation. So far, researchers were analyzing only one of these two aspects; in addition, only biochemical or imaging methods were used but never both, which were implemented in this study. Furthermore, this is the first and so far, the only study that analyzed the impact of BFRE on the clusters of differentiation such as PECAM1 or CD34 antibodies. Such a wide spectrum of measurements conducted on a homogeneous group of participants immediately after the BFRE and compared to control training gives a clear answer in the debate on the acute vascular response to exercises with restricted blood flow.

## 5. Conclusions

Based on the results of this study, we can assume that low-intensity BFR exercise performed by a professional with dedicated equipment, with precisely set pressure by well-fitted cuffs, is more efficient in the stimulation of angiogenesis compared to the same training performed without blood flow restriction. 

The results finally prove, without further concerns, that participants of BFRE benefit not only from an increase in muscle size and strength but also from a better blood supply by stimulation of the angiogenesis. In our opinion, the results are solid enough to introduce BFR exercising into a trial for patients in need of rehabilitation due to long-distance claudication caused by lower limb ischemia.

## Data Availability

All data are included in the study.
